# Inferring Evolutionary Process From Neuroanatomical Data

**DOI:** 10.3389/fnana.2018.00054

**Published:** 2018-07-27

**Authors:** Eric Lewitus

**Affiliations:** Institut de Biologie de l'ENS, Paris Sciences et Lettres Université, Paris, France

**Keywords:** macroevolution, phylogenetics, mammals, PGLS, likelihood-based methods

## Abstract

Brain evolution has interested neuroanatomists for over a century. These interests often fall on how free the brain is to evolve independently of the body, how free brain regions are to evolve independently of each other, and how different environmental and ecological factors affect the brain over evolutionary time. But despite major advances in phylogenetic methods, comparative neuroanatomists have tended to limit their macroevolutionary toolbox to regression-based analyses and ignored the scope of evolutionary process-based models at their disposal. This Review summarizes the history of comparative neuroanatomy and highlights the pitfalls of the methodologies traditionally used. It provides an overview of evolutionary process-based modeling approaches for investigating univariate and multivariate data, as well as more sophisticated methods that incorporate hypotheses about biotic and abiotic pressures that may drive brain evolution. The benefits of evolutionary process-based models, and shortcomings of regression-based ones, are illustrated with widely used neuroanatomical data. Ultimately, the intent of this Review is to be a guide for subsuming macroevolutionary methods not typically used in comparative neuroanatomy, in order to improve our understanding of how the brain evolves.

## Introduction

How the brain evolves is a question that has interested evolutionary biologists for over a century, likely because it is the same organ that is needed to ask the question. The long history of work investigating its answer has basically fallen into two camps: the ecological and social factors that underpin evolutionary changes in brain architecture (why the brain evolves); and the metabolic and functional constraints shaping those changes (how the brain evolves). Beginning in the nineteenth century, naturalists such as Dubois, Lapicque, and Snell recognized a consistent relationship between brain and body size, which was later formalized into the concept of allometry by Huxley and Teissier (1936). The concept that evolutionary changes in brain size are linked to changes in body size—and its extension, that there are coordinated evolutionary changes among different brain regions—has framed our understanding of how brains evolve (Jerison, 1975). Consequently, variation in brain size is generally interpreted in terms of how it deviates from allometric expectations. In the broadest terms, deviations are interpreted functionally, such that species with brains larger than expected for their body mass are interpreted as being more intelligent (Roth and Dicke, 2005); and, likewise, species with a brain region (e.g., the neocortex or striatum) larger than expected for their brain size are interpreted as having undergone adaptive selection for the functionality prescribed to that region. Our understanding of why the brain evolves, on the other hand, in an adaptive sense, assumes that changes in behavior are subsidized by changes in the neural substrates regulating that behavior. For example, the superior colliculus, a major visual region, is nearly 40 times smaller than predicted by allometry in the blind mole rat (Cooper et al., 1993), whereas the inferior colliculus, an auditory region, is several times larger than predicted by allometry in echolocating bats (Striedter, 2005). Of course, the identification and quantification of deviations from expectations depend on properly estimating the scaling exponent of the relationship, which can be tricky (Jerison, 1975; Harvey and Pagel, 1988; Grabowski et al., 2016), and can be additionally difficult to disentangle from evidence of convergene (Aristide et al., 2016; Mahler et al., 2017), which will affect evolutionary interpretations. These two camps have together amassed support for many hypotheses to explain interspecific variation in brain architecture. Among them are effects of diet and sociality on brain size (see Powell et al., 2017), differential enlargement of the prefrontal cortex in great apes (Smaers et al., 2017), and clade-specific trends in neuron number and density (Herculano-Houzel et al., 2014). However, the existence of support for so many (often incompatible) hypotheses underscores a problem (Dunbar and Shultz, 2017). This problem may be due in part to how we define traits and behaviors as they relate to the brain. But it is also certainly due to methodological shortcomings, and that is the focus of this review.

Support in comparative neuroanatomy is almost exclusively defined as correlational support, and analyses are largely restricted to regressions on continuous character states. While not wrong in themselves, these approaches are limited and rarely adequately assessed. The scope of ill-fitting evolutionary analyses in the field extend from run-of-the-mill mistakes in checking model assumptions (which are not uncommon in comparative biology generally) to misconceptions bordering on hostility to "unfortunate phylogenetic inferences" (Mota and Herculano-Houzel, 2016). Additionally, other types of phylogenetic approaches are almost never considered. While the compendium of existing work on brain evolution is not at all invalidated by this, it is valuable to understand what phylogenetic comparative methods (PCMs) can (and cannot) do, in order to ferret out exactly what this work says (and does not say) about how the brain evolves. Specifically, it is important to draw a distinction between methodological approaches that infer correlations between traits and those that infer the evolutionary processes driving traits to (co-)evolve.

We know that the brain is a highly integrated functional system that develops under evolutionarily conserved developmental constraints (Strausfeld and Hirth, 2013; Lewitus and Huttner, 2015). We also know that, as a consequence of this, the brain is not simply the sum of its individually evolving parts, but that it is comprised of modules that are shared across species (Goswami, 2007). Multivariate evolutionary models, which are becoming increasingly advanced and accessible (e.g., Bartoszek et al., 2012; Clavel et al., 2015), are key to investigating how these modules—whether they are genetic, cell-biological, or morphological—evolve differently across species. Ultimately, our goal should be aimed toward developing and applying increasingly sophisticated models that can account for the evolution of such an imposing, complex structure. Adopting an arsenal of process-based models strengthens our ability to investigate how the brain evolves by allowing us to consider explicit hypotheses of interspecific competition, environmental influences, trait covariation and causality, directional selection, and information on rates and modes of evolutionary change. In this paper, I review macroevolutionary theories for trait diversification and the advantages and disadvantages of both regression- and process-based PCMs. I provide examples of how regression-based analyses can lead to misleading or even spurious inferences of trait evolution. I analyse published neuroanatomical data using process-based modeling to illustrate the utility of this approach. Finally, I provide a blueprint for conducting macroevolutionary analyses on neuroanatomical data and outline how this might be used to incorporate data at different neurobiological scales. In full, it is important that this field takes advantage of the totality of available macroevolutionary methods.

## Phylogenetic comparative methods: a model-based approach

At its core, the evolutionary history of a trait is described by mode and rate of change. Does the cortex evolve faster than the cerebellum? Is there a slowdown in V1 evolution in primates? The mode and rate are defined by how (dis)similar trait values are among species over a given amount of time assuming a model of evolutionary change. As different models make different assumptions about data, infer different rates of change, and ultimately give different hypotheses about how traits evolved, model choice is absolutely critical.

### The univariate case

**Figure 1:**
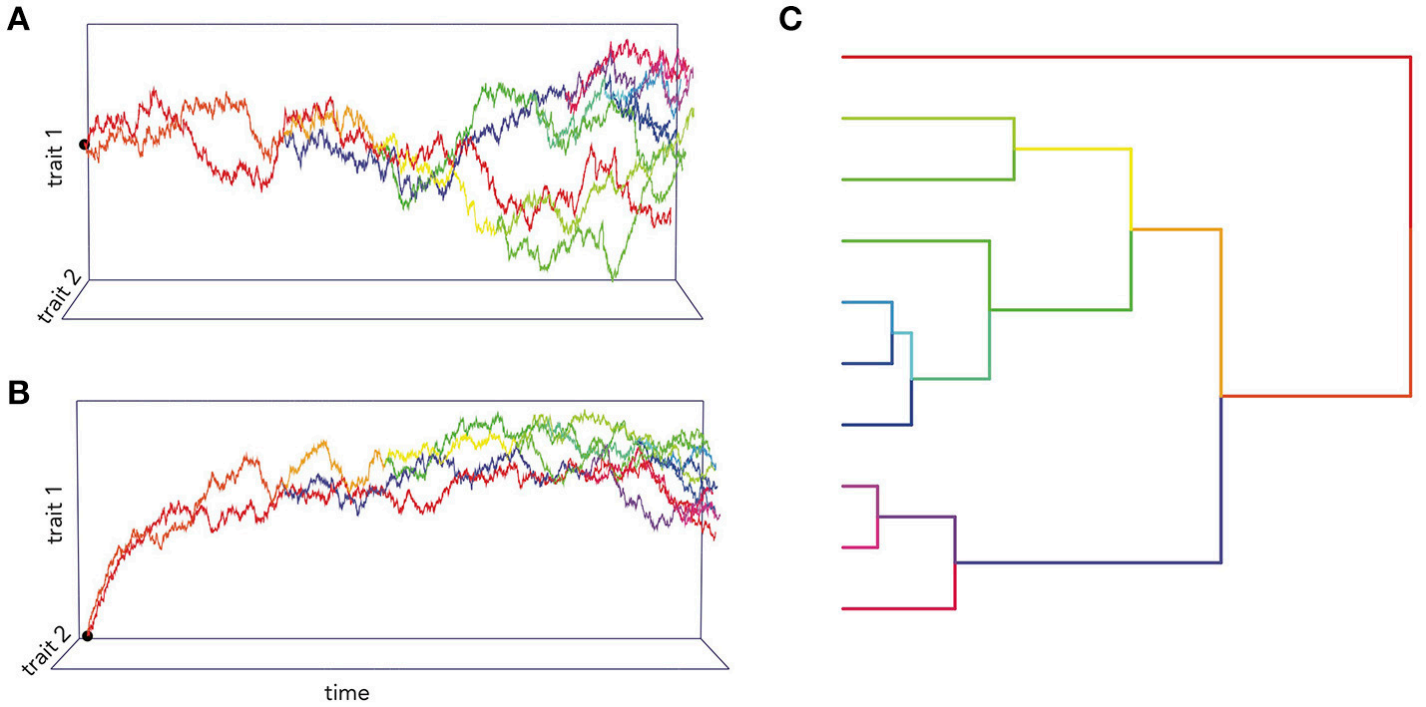
Simulations of two traits co-evolving under different processes. In **(A)**, the trait co-variance accrues linearly with time according to a BM process, while in **(B)** the traits are attracted toward an optimum value. **(C)** An example of phylogenetic trait changes over time.

**Figure 2:**
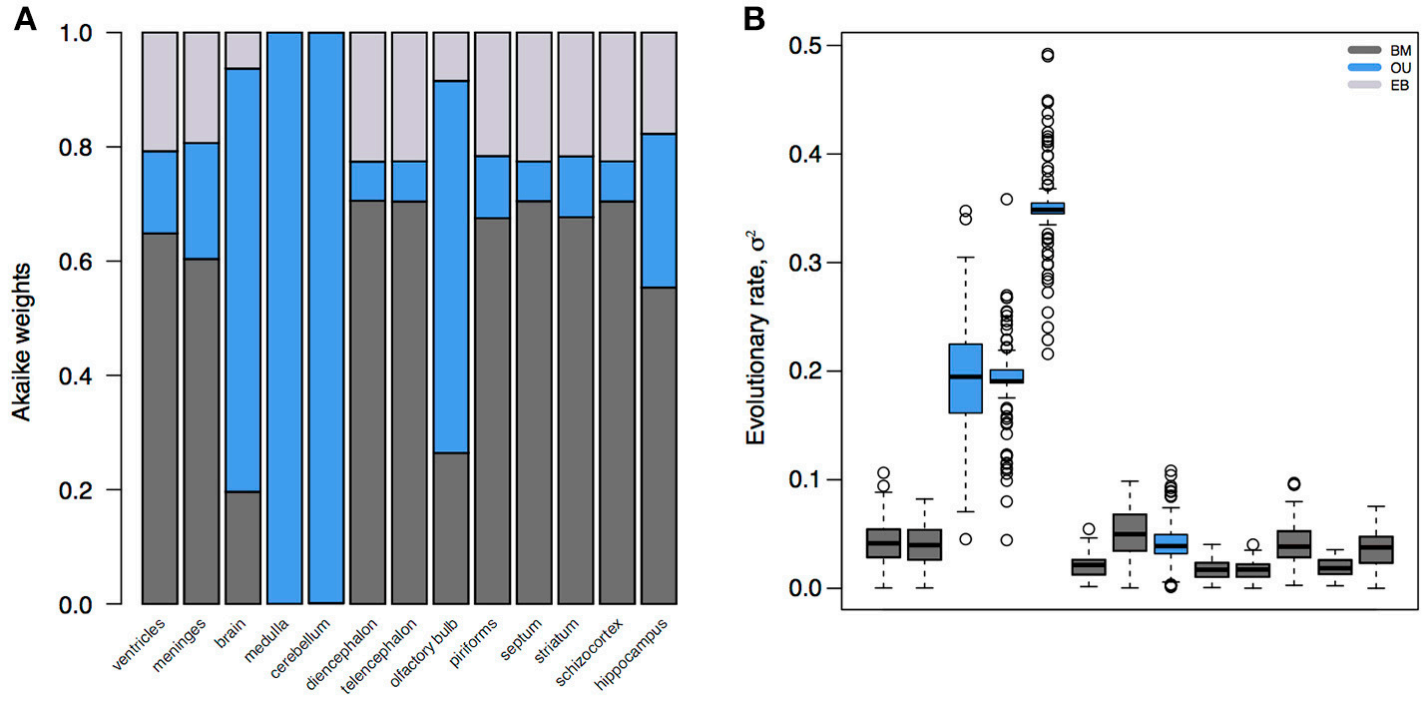
Model fits and parameter estimates for mammalian brain regions. **(A)** Akaike weights for BM, OU, and EB models fit to brain or brain region volume for mammalian species in Stephan et al. (1981). **(B)** Inferred evolutionary rate parameters for the best fit models. Note that, because the whole brain, medulla, cerebellum, and olfactory bulb are best supported by an OU model, their rate estimates are artifactually elevated compared to other regions. Model fits and parameter estimates computed using the *R* package *mvMORPH* (Clavel et al., 2015) on 100 posterior distributions of the mammalian phylogeny (Faurby and Svenning, 2015).

The standard model for trait evolution is Brownian motion (BM), which describes trait variance as a linear function of time. Under this model, the mean value of a trait can increase or decrease independently of its current state, as long as the net change is zero (Cavalli-Sforza and Edwards, 1967; Felsenstein, 1985). This is represented as *dY*_*t*_ = σ*dB*_*t*_ for a trait *Y* at time *t* with rate parameter σ. However, because species share an evolutionary history does not necessarily mean that trait variance accrues linearly with time (Figure 1). In fact, it is highly unlikely that all traits evolve according to a BM process; and changes in trait variance through time may not only be due to time-varying rates, but instead to shifts in evolutionary mode (Hunt, 2012; Slater, 2013). A modified version of the BM model is the Ornstein-Uhlenbeck (OU) model (Hansen, 1997; Butler and King, 2004), which includes an additional parameter to measure the tendency toward an optimal trait value, θ. This is represented as *dY*_*t*_ = −α(*Y*_*t*_−θ)*dt*+σ*dB*_*t*_, where α is the magnitude of stabilizing selection acting on the trait. Tendency toward an optimum can represent many things, including directional selection, response to an extrinsic factor, or developmental or functional constraint from an intrinsic factor. Likewise, the BM model can be relaxed to account for accelerating (AC) or decelerating (DC) rates of trait evolution through time (Blomberg et al., 2003). This is often used to test for signs of an early burst (EB) in the evolution of a trait, indicative of an adaptive radiation (Harmon et al., 2010). Given trait data on a phylogeny, we can calculate the likelihood of support for different models. We can then select the model with the most support (i.e., the best fit model) to infer the mode and rate of change of that trait across the phylogeny. In doing so, we can better understand the evolutionary process underlying the distribution of a trait across a clade, as well as compare processes and rates between clades. The dataset of Stephan et al. (1981), which is comprised of volumetric measurements for different brain regions for mammalian species, has been foundational for comparative neuroanatomy. But in the hundreds of analyses of this dataset, the measured brain volumes have always been assumed to evolve according to a BM process (Finlay and Darlington, 1995; Barton and Harvey, 2000) – or, in the case of the social brain hypothesis (Dunbar, 1992), not to have evolved according to any process at all. However, we can see that this is a bad assumption (Figure 2). Rather, whole brain, cerebellum, medulla oblongata, and olfactory bulb volume are better supported by OU processes, suggesting that in this subset of mammals different evolutionary processes are driving trait changes in different brain regions. This highlights the importance of finding the best model to explain the data under analysis, because the evolutionary story that is reconstructed for those data (e.g., an estimate of the brain volume of a clade's ancestor generated using ancestral state reconstruction under the best fit model) will necessarily change depending on the model fit to them.

### The multivariate case

**Figure 3:**
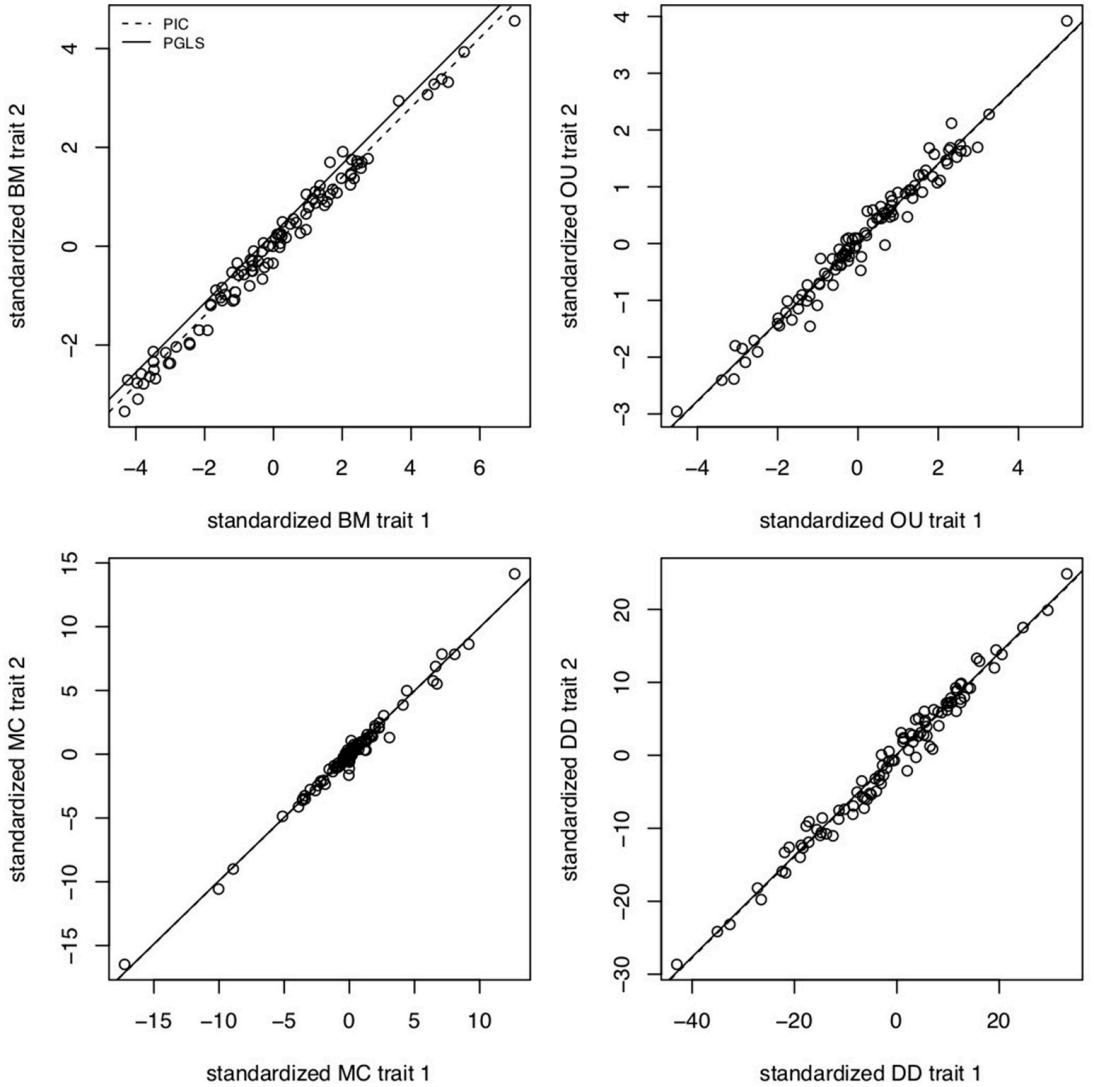
PGLS regressions often lead to spurious or uninformative results. Slope coefficients for PGLS and phylogenetic independent contrasts (PIC) on trait residuals using traits simulated under BM for σ^2^ = 0.1, OU for α = 5 and σ^2^ = 0.05, MC for *S* = −0.5 and σ = 0.05, and negative exponential diversity-dependence (DD) for rate parameter β = −0.5, equivalent to three times the phylogenetic half-life (Weir and Mursleen, 2013). Despite the different underlying processes, PGLS and PIC return coefficient estimates as if the processes were BM. Data simulated using mvMORPH (Clavel et al., 2015) and *RPANDA* (Morlon et al., 2016) and PGLS and PIC fits computed with *ape* (Paradis et al., 2004).

**Figure 4:**
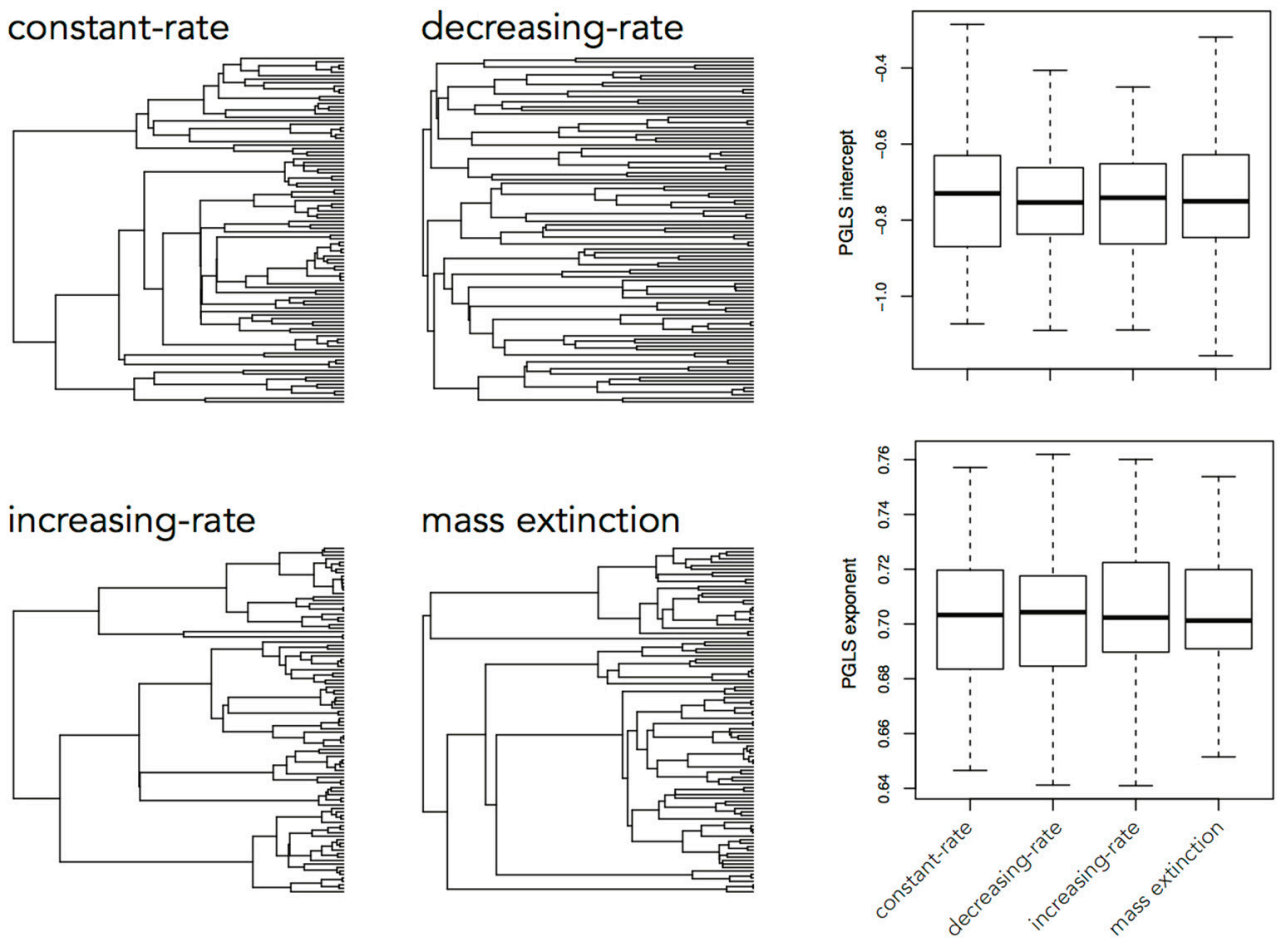
Slope coefficients and intercept estimates for PGLS analyses on trait data simulated using a BM process on a constant-rate birth-death tree, but recovered using trees simulated under different processes. A trait simulated under a BM process on one tree should not be similarly distributed across a tree simulated under a completely different process and therefore the returned slope estimate should be different.

The brain is a complex organ with many interacting parts. Those parts tend to be developmentally or functionally integrated, which can result in co-evolution due to genetic covariance or correlated selection (Armbruster et al., 2014). So a frequent question in comparative neuroanatomy is how two (or more) traits co-evolve. The typical analytical approach for determining correlations between two variables is ordinary least squares (OLS) regression, but this assumes statistically independent data. Because species share an evolutionary history, they are not independent data points and therefore OLS regression is not typically appropriate [but see Revell (2010)]. Rather, the most popular method for measuring correlations between species traits is phylogenetic generalized least squares (PGLS; a generalization of phylogenetic independent contrasts) (Grafen, 1989; Garland and Ives, 2000; Blomberg et al., 2012). In its simplest (and most common) form, PGLS assumes a BM process; but this comes with certain theoretical and statistical limitations. Specifically, as traits are not expected to evolve uniformly across a phylogeny, especially when that phylogeny is large, PGLS regressions assuming a BM model show high type I error rates (Revell, 2010). Consequently, the residuals of trait values generated by different processes simulated on the same tree are well fit by a PGLS regression assuming a BM process, giving no indication of the actual underlying evolutionary process (Figure 3). This would lead to the spurious conclusion that the traits are correlated under a BM process. Similarly, PGLS regression analyses on identical tip data for trees simulated under different evolutionary processes return statistically identical scaling coefficients, making no distinction between considerably different evolutionary scenarios (Figure 4). Again, this analysis masks the true and more complex evolutionary history of these traits. Moreover, when there is uncorrelated residual error in the dependent variable, PGLS performs even worse than OLS (Revell, 2010). This is likely due to the (overlooked) fact that PGLS does not measure evolutionary covariance between traits, but rather the evolutionary covariance of one trait with respect to the tip values of another and therefore is not sensitive to trait covariance through time. In other words, any evidence of correlation cannot (on its own) be interpreted as adaptive, despite claims to the contrary (Nunn and Barton, 2001). These theoretical and statistical pitfalls should be considered when choosing an approach for modeling multivariate trait evolution.

Fortunately, a number of methods have been developed to detect evidence of adaptive evolution in co-evolving traits (e.g., Revell and Harmon, 2008; Revell and Collar, 2009; Hadfield, 2010; Eastman et al., 2011), which measure how traits affect each other's evolution. As mentioned, one of the most frequently debated topics in comparative neuroanatomy is the evolutionary relationship between brain and body size. However, this debate has largely been focused on how species are sampled or how brain size is measured, but has not been concerned with how the relationship is modeled, even though this relationship is significantly affected by model assumptions. Recently, Grabowski et al. (2016) showed that brain size allometry in mammals is best explained by a model (Hansen and Bartoszek, 2012) that explicitly allows body size (a predictor variable) to cause changes in the variance of brain size (a response variable). In addition to highlighting the spurious affects of using the wrong model, this result provides a more meaningful explanation for the nature of brain-body allometry that goes beyond a simple scaling coefficient. More broadly, there have been many advances to estimating correlated rates of change using the evolutionary rate matrix (Revell and Harmon, 2008; Bartoszek et al., 2012; Beaulieu et al., 2012; Clavel et al., 2015), which provides a framework for inferring the process behind evolutionary change in correlated traits. An evolutionary variance-covariance matrix is especially advantageous for studying correlated evolution, because it allows for estimating rates for individual traits as well as covariance between pairs of traits, which is additionally useful for testing different models of trait evolution and detecting shifts in rates leading to specific clades. To take the relationship between the cortex (whole brain minus cerebellum) and cerebellum as an example, which has been measured numerous times elsewhere using PGLS and derived some correlational support under a BM process (e.g., Barton and Harvey, 2000; Herculano-Houzel, 2010; Barton and Venditti, 2014), we can estimate the relative support of interacting and non-interacting BM and OU processes. In doing so, we are directly comparing models in which rates of change in the cortex and cerebellum co-evolve (with and without stabilizing selection) against those in which cortical and cerebellum volume evolve independently of one another. We find that the co-evolution of cortical and cerebellum volume in a subset of mammals (Stephan et al., 1981) is best explained by a process in which both evolve toward optima under stabilizing selection (Δ*AICc* = 11.68), while exerting negative influences on each other over time (α = −0.489). To take another example, of neuron and glia cell numbers in the mammalian cortex (Herculano-Houzel, 2011), we find this relationship is best supported by an interacting BM process (Δ*AICc* = 9.02), wherein both cortical neuron and glia cell number evolve under a BM model, but also exert positive influences on each other through time (σ^2^ = 0.045). (Note that here σ^2^ represents evolutionary covariance.) In both cases, the evolution of the two traits – cortical and cerebellum volume and cortical neuron and glia number – can be better explained by their covariance through time rather than by treating them as independently evolving, which is contrary to how the data were treated in previous analyses.

### Hypothesis-based approaches

**Figure 5:**
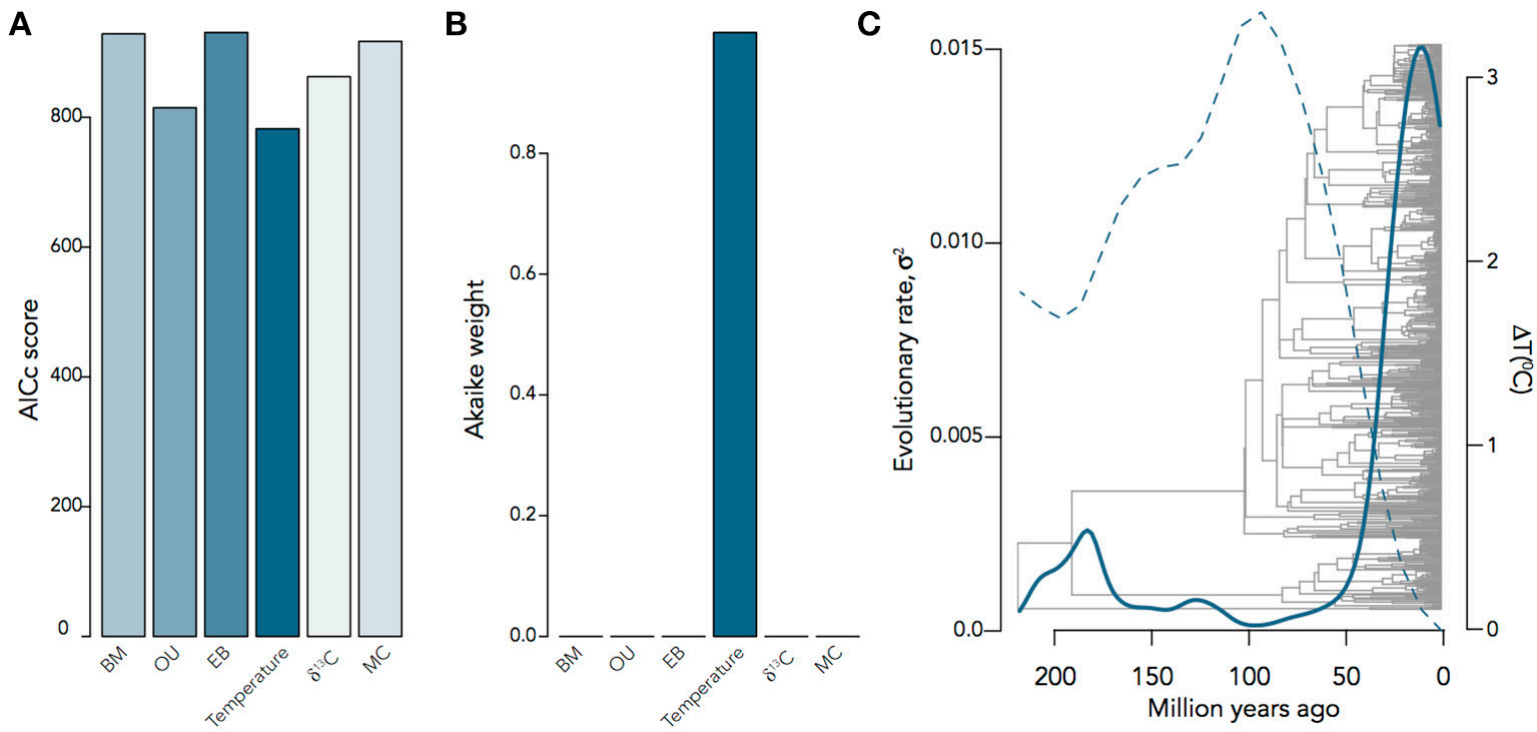
Inferring the evolutionary process underlying mammalian EQ evolution. **(A)** AIC scores and **(B)** Akaike weights for BM, OU, EB, two environment-dependent models, and the MC model fit to mammalian EQ data from Boddy et al. (2012). **(C)** Temperature-dependent evolutionary rate through time of EQ variance in mammals, showing that variation in EQ (solid) changes as an inverse exponential function of temperature (dashed): σ^2^(*t*) = 0.05*e*^−9.13*T*(*t*)^. Environmental curves from Mayhew et al. (2008); Hannisdal and Peters (2011).

It is often the case that we want to know not only the evolutionary mode and tempo of a trait or even the correlated evolution of two traits, but what other factors were involved in driving evolutionary trait change. Any number of factors can affect the rate of change of a trait, including habitat, interspecific competition, or the global environment; and a number of models have been developed to investigate the relative importance of each of these factors. The environment-dependent model of trait evolution (Clavel and Morlon, 2017) measures the rate of change of a trait or the covariance of traits as a function of some time-varying factor (e.g., temperature). The matching competition (MC) model (Nuismer and Harmon, 2015; Drury et al., 2016) can be used to estimate the effect interspecific competition has on trait variance through time by computing how changes of trait variance in one direction drive trait variance in the opposite direction among species within a clade. These models do not simply estimate the effects of the environment or interspecific interactions on rates of trait evolution, but by estimating likelihoods for each model they can be compared to each other and the trait models above. We can see, for example, that encephalization quotient (EQ) evolution in mammals is best supported by a model of temperature-dependence (Δ*AICc* = 26.06) over BM, OU, EB, MC, or δ^13^*C*−dependence (Figure 5), where EQ has a negative dependency on temperature through time. By using these hypothesis-based models, we can add another layer to our understanding of not only the mode and tempo of brain evolution, but the environmental and ecological selection pressures driving them.

There are, of course, a number of hypothesis-based models that can be implemented beyond those tested here. These include an interclade interaction model (Manceau et al., 2016), which can be particularly useful in highly divergent but ecologically connected clades, such as carnivores and ungulates, where a predator-prey relationship may have driven an evolutionary arms race in cortical evolution. State-dependent speciation and extinction (SSE) models, which estimate the effects of changes in trait variance with shifts in phylogenetic diversification (Maddison et al., 2007) and can be especially useful in disentangling shifts in rates of trait change from shifts in speciation or extinction rates. Models that incorporate fossil data (on, for example, endocranial volume) have also been developed to guide evolutionary inference using the fossil record (Heath et al., 2014). Perhaps the most widespread models are rate-shifting models, which estimate shifts in rates of trait evolution along certain branches or in certain clades (Rabosky, 2014). Many comparative analyses assume clade-specific patterns *a priori* and then derive different regression coefficients for those clades as evidence of differences in patterns (e.g., Herculano-Houzel, 2011; Neves et al., 2014; Mota and Herculano-Houzel, 2015). Of course, clades are hypotheses themselves and constraining analyses by such assumptions not only limits the questions we can ask (and answer), but also biases analyses toward finding clade-specific patterns. Rate-shifting models, therefore, are an unbiased approach to determining whether there are indeed shifts in trait (co-)variances leading to certain clades. In total, the inclusion of these models can provide a much larger framework for inferring evolutionary scenarios leading to changes in neuroanatomy and estimating exactly the effect of those scenarios on neuroanatomical differences across species.

### A checklist for applying model-based PCMs to neuroanatomical data

The model one chooses to use in an analysis is as much an hypothesis as the biological question under analysis. Therefore, there are a number of questions that should be asked in the data analysis pipeline. Equally importantly, these questions (and their qualifications) should be stated explicitly in any analysis, in order to help the reader understand why a certain model was chosen and what assumptions (and limitations) should be associated with the result. Firstly, how good is our phylogeny? Approximately no trees are perfectly accurate, and so to account for a lack of confidence in topology and branch-lengths, it is best practice to use a distribution of phylogenies and sum results over all of them. This can be done, for example, using Bayesian inference to take a posterior probability distribution of a phylogenetic reconstruction (Bouckaert et al., 2014). Secondly, which models are appropriate for our data? It is, of course, possible to simply run the gamut of available trait models and see what sticks (best), however, this makes it increasingly difficult to interpret results in a meaningful way. In the most basic sense, a phylogenetic analysis should be treated like an experiment, leveraging a hypothesis-driven model (e.g., OU or MC) against a null one. Commonly used methods for estimating the presence of any phylogenetic signal include Blomberg's *K* (Blomberg et al., 2003), Moran's *I* (Münkemüller et al., 2012), and Pagel's λ (Pagel, 1999). [The latter, however, should be used with care: smaller trees are biased toward near-zero λ irrespective of a true phylogenetic signal; and traits with a complex evolutionary history can return spurious λ values (Boettiger et al., 2012)]. Thirdly, do we have enough power to derive a meaningful result? This, too, is a quantification of certainty in results and can be assessed by computing Akaike weights of different models, in order to give a sense of how much better one model is compared to another. If all models show comparable Akaike weights, then we may not have enough power to accurately assess the true evolutionary process (e.g., the phylogeny is too small) or we may not have included the correct model in our analysis. Finally, how many ways can we interpret our results? Because data collection, phylogeny reconstruction, and model choice are all hypotheses with associated assumptions, the derived results of any analysis are necessarily a product of those assumptions. Therefore, it is crucial to report not only the statistical confidence of results, but also the conceptual confidence based on the limitations imposed by every analytical step.

### Connecting neurobiological processes to macroevolutionary processes

So much of comparative neuroanatomy has been devoted to characterizing cell-biological or molecular differences between species (Yeung et al., 2017). This has helped find, for example, similarities in cortical pyramidal neurons across primates (Sherwood et al., 2003), differences in glia-neuron ratios in primates and carnivores (Lewitus et al., 2012), and modifications to visual pathways in monkeys and humans (Preuss and Coleman, 2002). There is also, of course, a monumental body of experimental work demonstrating the functionality of differences in gene architecture and neural cell morphology in model organisms, from the characterization of neural progenitor diversity in primates (Betizeau et al., 2013) to the identification of human-specific brain expansion genes (Florio et al., 2015). Ecological pressures act on behaviors, which lead to changes in developmental programming of the circuitry regulating those behaviors, and those changes are underwritten by selection on genes. Yet, these scales of biological phenomena are rarely integrated into phylogenetic studies of brain evolution. The few exceptions, including work on the role of microcephaly genes in primate brain evolution (Montgomery and Mundy, 2012) and the adaptiveness of self-renewing bipolar progenitors in the evolution of cortical folding (Lewitus et al., 2014), have shown the potential of integrating these scales to fine-tune our understanding of how the brain evolved. Future studies can take advantage of the advancing approaches devoted to identifying and characterizing modularity Goswami and Finarelli (2016) and co-evolution in multiple neuroanatomical traits (Morlon et al., 2016), which can be used to investigate how functionally integrated aspects of the brain evolved. Model-based phylogenetics provides a framework for investigating the macroevolutionary processes governing changes in neuroanatomy across species and can become a useful tool for investigating the social and environmental factors that have shaped the brain at the morphological and molecular level.

## Author contributions

This work was conceived and executed by EL.

### Conflict of interest statement

The author declares that the research was conducted in the absence of any commercial or financial relationships that could be construed as a potential conflict of interest.
